# On the Treatment of Chorea

**Published:** 1811-03

**Authors:** 


### *To the Editors of the Medical and Physical Journal.*

GENTLEMEN,

HAVING for the last fifty years been actively engaged in the practice of medicine, and read and studied all the theories and new plans of curing diseases, nothing has more surprised me, than the *directly opposite means* recommended for the cure of many, particularly scalds and burns, gout, chorea sancti viti, &c. &c. Professional gentlemen of the highest talents are still at war, respecting their opposite and favourite plans, and have left their humbler brethren at a loss, which to pursue, and occasioned many to adhere to that mode of practice from which they have experienced the best results. I have seen numerous cases of chorea s. v. and having invariably succeeded by the tonic and stimulating plan, I was the more surprised to see purging advised in one of your late valuable Journals, with a case or more cured thereby. Without at all doubting the fact, I beg leave to relate, as shortly as possible, two or three cases recently cured by the tonic plan.

A young married woman, of a feeble habit, was brought to me in a cart, from Hoxne in Suffolk; she had many months been affected with chorea, and rendered unable to look after her family. She looked like an ideot, was in a very weak state, with spasmodic affections in different muscles, particularly in those of her neck and arms.

The complaint was preceded by a lying-in, accompanied with a great loss. She had been taking medicines, and was believed by her husband and neighbours to be in "bad handling," the Suffolk vulgarism for witchcraft.

I encouraged my patient in the belief of my power to lay the evil spirit, if she would follow my advice to have her head shaved and plaistered, and take a gentle emetic, &c. which she agreed to do; after which a moderate dose of calomel was directed to clear the bowels. Tonic pills with ferrum, zinc vit. opium, camph. and an aromatic, were given night and morning, and a cordial decoct. of cort. cascar. with vitriolic acid, compleated her cure in a short time; a second blister was found necessary to apply to her head, which part of the plan is most necessary to attend to, and without which, or a strong rubefacient plaister, there is much less expectation of success.

A gentleman consulted me for his daughter, a fine girl of about nine years of age, who had been under the care of two very able medical gentlemen several weeks, for chorea; from whom whom I was favored with a letter, stating the plans they had pursued, and particularly their having depended on *purging*, as recommended by Dr. Hamilton, of Edinburgh, but were sorry to say, not only without benefit, but they found their patient getting worse; so said her father, observing she could neither walk or stand, and had entirely lost the use of one arm.

The complaint seemed to be induced by her having been kept poorly at a boarding school, and growing unusually fast; this made the tonic, restorative and stimulating plan, evidently necessary. Her head was directed to be shaved, a *strong* rubefacient plaister applied and to be repeated; a calomel purge to clear the bowels, after which steel, zinc, cortex, &c. as in the first case. These remedies so far recovered her, with the assistance of a generous regimen, that in ten days she could walk with safety and feed herself, with her before useless arm, to the surprise of her disconsolate parents, who brought her down ten miles rejoicing to show her me. Cold sea-bathing was found of great service towards completing her cure.

Mr. Tydiman, a fine young farmer of nineteen, from growing very fast, has been attacked with chorea, especially in his arms; he is now under my directions, and has found immediate benefit by shaving and blistering his head, taking medicines as above directed and living on a generous diet. I could bring forward numerous other cases of chorea, successfully treated by the above general plan, modifying it according to circumstances.

I have also frequently seen insanity *sine febre*, proceeding from grief, lowness of spirits, and debility, give way to *blistering the head* and the tonic stimulating plan, as above recommended.

I am, Gentlemen,

Your obliged Servant,

VERAX.

